# Supplementary material for: Direct On-Chip Diagnostics of Streptococcus bovis/Streptococcus equinus Complex in Bovine Mastitis Using Bioinformatics-Driven Portable qPCR
Source: Biomolecules. 2024 Dec 18;14(12):1624. doi: 10.3390/biom14121624 (PMC11726764; doi:10.3390/biom14121624)
Supplement: Supplementary file 1 [file biomolecules-14-01624-s001.zip › biomolecules-3334825-supplementary.pdf]

Table S1. Reference strains with corresponding Ct values demonstrating specificity of portable PCR using specific primers

Species	Reference no.[1]	Ct value of portable PCR with specific primers[2]			
		S. alactolyticus	*S. equinus*	*S. gallolyticus*	*S. lutetiensis*
Streptococcus alactolyticus	KVCC 13886	12.97 ± 0.03	ND	ND	ND
Streptococcus equinus	KVCC 13808	ND	15.05 ± 0.03	ND	ND
Streptococcus gallolyticus subsp. *gallolyticus*	KVCC 13794	ND	ND	14.27 ± 0.05	ND
Streptococcus gallolyticus subsp. *macedonicus*	KVCC 13851	ND	ND	14.23 ± 0.05	ND
Streptococcus gallolyticus subsp. *pasteurianus*	KVCC 13789	ND	ND	15.44 ± 0.04	ND
Streptococcus lutetiensis	KVCC 13788	ND	ND	ND	13.60 ± 0.04
Streptococcus cristatus	KACC 13831	ND	ND	ND	ND
Streptococcus equi subsp. *equi*	KACC 13809	ND	ND	ND	ND
Streptococcus equi subsp. *ruminatorum*	KACC 13796	ND	ND	ND	ND
Streptococcus mutans	KACC 16833	ND	ND	ND	ND
Streptococcus salivarius subsp. *thermophilus*	KACC 11857	ND	ND	ND	ND
Streptococcus sanguinis	KACC 11301	ND	ND	ND	ND
Enterococcus faecalis	KCTC 3206	ND	ND	ND	ND
Enterococcus faecalis	KACC 11859	ND	ND	ND	ND
Enterococcus faecium	KACC 11954	ND	ND	ND	ND
Enterococcus faecium	KCTC 13225	ND	ND	ND	ND
Enterococcus gilvus	KACC 13847	ND	ND	ND	ND
Pediococcus acidilactici	KACC 12307	ND	ND	ND	ND
Pediococcus argentinicus	KACC 16352	ND	ND	ND	ND
Pediococcus cellicola	KACC 12299	ND	ND	ND	ND
Pediococcus claussenii	KCTC 3811	ND	ND	ND	ND
Pediococcus damnosus	KACC 12309	ND	ND	ND	ND
Pediococcus ethanolidurans	KACC 15276	ND	ND	ND	ND
Pediococcus inopinatus	KACC 12308	ND	ND	ND	ND
Pediococcus parvulus	KACC 12310	ND	ND	ND	ND
Pediococcus pentosaceus	KACC 12311	ND	ND	ND	ND
Pediococcus stilesii	KACC 12300	ND	ND	ND	ND
Staphylococcus arlettae	KACC 13254	ND	ND	ND	ND

Staphylococcus capitis	KACC 13242	ND	ND	ND	ND
Staphylococcus caprae	KCTC 3583	ND	ND	ND	ND
Staphylococcus carnosus	KACC 13190	ND	ND	ND	ND
Staphylococcus cohnii	KACC 13237	ND	ND	ND	ND
Staphylococcus edaphicus	KCTC 43531	ND	ND	ND	ND
Staphylococcus epidermidis	KACC 13234	ND	ND	ND	ND
Staphylococcus equorum	KACC 13255	ND	ND	ND	ND
Staphylococcus haemolyticus	KACC 18729	ND	ND	ND	ND
Staphylococcus hominis	KACC 13712	ND	ND	ND	ND
Staphylococcus intermedius	KCTC 3344	ND	ND	ND	ND
Staphylococcus muscae	KCTC 3576	ND	ND	ND	ND
Staphylococcus nepalensis	KACC 16177	ND	ND	ND	ND
Staphylococcus pasteuri	KCTC 13173	ND	ND	ND	ND
Staphylococcus saprophyticus	KACC 13231	ND	ND	ND	ND
Staphylococcus simulans	KACC 13241	ND	ND	ND	ND
Staphylococcus succinus	KACC 13202	ND	ND	ND	ND
Staphylococcus ureilyticus	KACC 13173	ND	ND	ND	ND
Staphylococcus warneri	KACC 13240	ND	ND	ND	ND
Staphylococcus xylosus	KACC 13239	ND	ND	ND	ND

[1]ND, Not detected

[2]KVCC, Korea Veterinary Culture Collection; KACC, Korean Agricultural Culture Collection; KCTC, Korean Collection for Type Cultures

Table S2. List of SBSEC genomes used for pangenome analysis to identify genetic markers

Organism name	Size (bp)	Assembly level	Assembly accession
Streptococcus alactolyticus	1,750,188	Contig	GCF_009695625.1
Streptococcus alactolyticus	1,698,167	Contig	GCF_012277075.1
Streptococcus alactolyticus	1,674,970	Contig	GCF_016899875.1
Streptococcus alactolyticus	1,675,446	Contig	GCF_021531835.1
Streptococcus alactolyticus	1,735,065	Contig	GCF_021531925.1
Streptococcus alactolyticus	1,699,682	Contig	GCF_026011995.1
Streptococcus alactolyticus	1,832,198	Complete	GCF_027474505.1
Streptococcus equinus	1,741,734	Scaffold	GCF_000187265.1
Streptococcus equinus	1,843,423	Contig	GCF_000421425.1
Streptococcus equinus	1,786,673	Scaffold	GCF_000424045.1
Streptococcus equinus	1,919,820	Scaffold	GCF_000702105.1
Streptococcus equinus	1,846,241	Scaffold	GCF_000731085.1
Streptococcus equinus	1,841,890	Contig	GCF_000747195.1
Streptococcus equinus	1,954,216	Contig	GCF_000747205.1
Streptococcus equinus	1,930,741	Contig	GCF_000964315.1
Streptococcus equinus	1,935,083	Contig	GCF_004525775.1
Streptococcus equinus	1,926,856	Contig	GCF_006538945.1
Streptococcus equinus	1,911,874	Complete	GCF_009758075.1
Streptococcus equinus	1,997,064	Complete	GCF_009758095.1
Streptococcus equinus	1,936,555	Complete	GCF_014041875.1
Streptococcus equinus	2,083,084	Scaffold	GCF_000146405.1
Streptococcus equinus	1,867,430	Contig	GCF_015059535.1
Streptococcus equinus	1,904,424	Chromosome	GCF_016906125.1
Streptococcus equinus	1,994,270	Complete	GCF_022810625.1
Streptococcus equinus	1,884,420	Complete	GCF_024732405.1
Streptococcus equinus	1,870,038	Contig	GCF_028823545.1
Streptococcus equinus	1,855,301	Contig	GCF_028823555.1
Streptococcus equinus	1,825,979	Scaffold	GCF_900099885.1
Streptococcus equinus	1,799,853	Contig	GCF_900100035.1
Streptococcus equinus	1,863,305	Scaffold	GCF_900100215.1
Streptococcus equinus	1,962,621	Scaffold	GCF_900101445.1
Streptococcus equinus	1,887,161	Contig	GCF_900101715.1
Streptococcus equinus	1,797,051	Scaffold	GCF_900102715.1
Streptococcus equinus	2,042,259	Scaffold	GCF_900103355.1
Streptococcus equinus	1,875,611	Contig	GCF_900104225.1
Streptococcus equinus	1,909,978	Contig	GCF_900106895.1
Streptococcus equinus	1,931,095	Contig	GCF_900109105.1
Streptococcus equinus	1,820,620	Scaffold	GCF_900109395.1
Streptococcus equinus	1,875,780	Scaffold	GCF_900109415.1
Streptococcus equinus	1,827,295	Scaffold	GCF_900110365.1
Streptococcus equinus	1,864,122	Scaffold	GCF_900110935.1
Streptococcus equinus	1,878,198	Scaffold	GCF_900112385.1
Streptococcus equinus	1,912,748	Scaffold	GCF_900113235.1
Streptococcus equinus	1,967,362	Scaffold	GCF_900114525.1
Streptococcus equinus	1,912,510	Scaffold	GCF_900115895.1
Streptococcus equinus	2,021,307	Contig	GCF_900199575.1

Streptococcus equinus	1,806,069	Contig	GCF_900459295.1
Streptococcus equinus	1,759,508	Contig	GCF_900459945.1
Streptococcus equinus	1,873,522	Complete	GCF_900636465.1
Streptococcus equinus	1,781,995	Complete	GCF_900636565.1
Streptococcus equinus	1,867,673	Complete	GCF_901543475.1
Streptococcus equinus	1,741,734	Scaffold	GCF_902374225.1
Streptococcus gallolyticus	2,350,911	Complete	GCF_000027185.1
Streptococcus gallolyticus	2,377,209	Complete	GCF_000203195.1
Streptococcus gallolyticus	2,362,241	Complete	GCF_000270145.1
Streptococcus gallolyticus	2,639,074	Contig	GCF_000723985.1
Streptococcus gallolyticus	2,311,421	Contig	GCF_000949245.1
Streptococcus gallolyticus	2,530,481	Scaffold	GCF_001578695.1
Streptococcus gallolyticus	2,548,002	Scaffold	GCF_001579135.1
Streptococcus gallolyticus	2,332,206	Scaffold	GCF_001658075.1
Streptococcus gallolyticus	2,330,998	Scaffold	GCF_001703415.1
Streptococcus gallolyticus	2,492,900	Complete	GCF_002000985.1
Streptococcus gallolyticus	2,184,549	Scaffold	GCF_003337175.1
Streptococcus gallolyticus	2,159,822	Scaffold	GCF_003433685.1
Streptococcus gallolyticus	2,155,233	Scaffold	GCF_003434265.1
Streptococcus gallolyticus	2,191,206	Chromosome	GCF_012273095.1
Streptococcus gallolyticus	2,258,003	Complete	GCF_013267695.1
Streptococcus gallolyticus	2,246,969	Scaffold	GCF_000146525.1
Streptococcus gallolyticus	2,052,496	Complete	GCF_001477575.1
Streptococcus gallolyticus	2,131,986	Contig	GCF_001481475.1
Streptococcus gallolyticus	2,258,003	Complete	GCF_019021805.1
Streptococcus gallolyticus	2,544,083	Contig	GCF_021474745.2
Streptococcus gallolyticus	2,169,656	Scaffold	GCF_022427105.1
Streptococcus gallolyticus	2,222,864	Scaffold	GCF_023109635.1
Streptococcus gallolyticus	2,406,997	Scaffold	GCF_023469885.1
Streptococcus gallolyticus	2,302,966	Contig	GCF_024125525.1
Streptococcus gallolyticus	2,384,548	Contig	GCF_024125535.1
Streptococcus gallolyticus	2,273,386	Contig	GCF_024580595.1
Streptococcus gallolyticus	2,243,104	Scaffold	GCF_026783185.1
Streptococcus gallolyticus	2,313,793	Scaffold	GCF_026783245.1
Streptococcus gallolyticus	2,403,397	Scaffold	GCF_026783265.1
Streptococcus gallolyticus	2,034,639	Contig	GCF_026783285.1
Streptococcus gallolyticus	2,247,471	Contig	GCF_026783305.1
Streptococcus gallolyticus	2,341,429	Scaffold	GCF_026783325.1
Streptococcus gallolyticus	2,234,500	Scaffold	GCF_026783345.1
Streptococcus gallolyticus	2,236,602	Contig	GCF_026783355.1
Streptococcus gallolyticus	2,242,356	Scaffold	GCF_026783385.1
Streptococcus gallolyticus	2,134,750	Scaffold	GCF_026783405.1
Streptococcus gallolyticus	2,353,094	Scaffold	GCF_026783425.1
Streptococcus gallolyticus	2,341,635	Scaffold	GCF_026783445.1
Streptococcus gallolyticus	2,232,060	Scaffold	GCF_026783455.1
Streptococcus gallolyticus	2,341,290	Contig	GCF_026783485.1
Streptococcus gallolyticus	2,355,468	Scaffold	GCF_026783505.1
Streptococcus gallolyticus	2,211,625	Scaffold	GCF_026783525.1
Streptococcus gallolyticus	2,341,660	Scaffold	GCF_026783565.1

Streptococcus gallolyticus	2,341,963	Scaffold	GCF_026783575.1
Streptococcus gallolyticus	2,392,629	Complete	GCF_027474865.2
Streptococcus gallolyticus	2,176,773	Scaffold	GCF_900101135.1
Streptococcus gallolyticus	2,175,210	Scaffold	GCF_900103105.1
Streptococcus gallolyticus	2,182,305	Scaffold	GCF_900107905.1
Streptococcus gallolyticus	2,192,837	Scaffold	GCF_900109835.1
Streptococcus gallolyticus	2,178,625	Scaffold	GCF_900111215.1
Streptococcus gallolyticus	2,370,282	Scaffold	GCF_900112565.1
Streptococcus gallolyticus	2,368,938	Scaffold	GCF_900116655.1
Streptococcus gallolyticus	2,493,581	Complete	GCF_900475715.1
Streptococcus infantarius	1,925,187	Scaffold	GCF_000154985.1
Streptococcus infantarius	1,958,742	Complete	GCF_016127275.1
Streptococcus infantarius	1,907,727	Scaffold	GCF_018499205.1
Streptococcus infantarius	1,916,150	Scaffold	GCF_026782965.1
Streptococcus infantarius	1,883,350	Scaffold	GCF_026783005.1
Streptococcus infantarius	1,838,625	Scaffold	GCF_027664625.1
Streptococcus infantarius	1,769,009	Scaffold	GCF_027674615.1
Streptococcus infantarius	1,841,133	Scaffold	GCF_027674625.1
Streptococcus infantarius	1,773,864	Scaffold	GCF_027674965.1
Streptococcus infantarius	1,783,593	Scaffold	GCF_027675065.1
Streptococcus infantarius	1,844,703	Scaffold	GCF_027675125.1
Streptococcus infantarius	1,818,858	Scaffold	GCF_027675225.1
Streptococcus infantarius	1,836,607	Scaffold	GCF_027675305.1
Streptococcus infantarius	1,801,660	Scaffold	GCF_027675345.1
Streptococcus infantarius	1,795,654	Scaffold	GCF_027675365.1
Streptococcus infantarius	1,827,969	Scaffold	GCF_027675395.1
Streptococcus infantarius	1,837,865	Scaffold	GCF_027675425.1
Streptococcus infantarius	1,836,299	Scaffold	GCF_027675525.1
Streptococcus infantarius	1,811,421	Scaffold	GCF_027675545.1
Streptococcus infantarius	1,855,416	Scaffold	GCF_027675655.1
Streptococcus infantarius	1,776,632	Scaffold	GCF_027675685.1
Streptococcus infantarius	1,841,825	Scaffold	GCF_027675965.1
Streptococcus infantarius	1,919,076	Scaffold	GCF_027676245.1
Streptococcus infantarius	1,912,587	Scaffold	GCF_027676405.1
Streptococcus infantarius	1,893,755	Scaffold	GCF_027676605.1
Streptococcus infantarius	1,775,373	Scaffold	GCF_027676685.1
Streptococcus infantarius	1,966,072	Scaffold	GCF_027723585.1
Streptococcus infantarius	1,806,980	Scaffold	GCF_027723645.1
Streptococcus infantarius	1,782,490	Scaffold	GCF_027723865.1
Streptococcus infantarius	1,792,934	Scaffold	GCF_027723925.1
Streptococcus infantarius	1,925,187	Scaffold	GCF_902373735.2
Streptococcus lutetiensis	1,975,547	Complete	GCF_000441535.1
Streptococcus lutetiensis	1,801,107	Scaffold	GCF_001578785.1
Streptococcus lutetiensis	1,887,340	Scaffold	GCF_003470165.1
Streptococcus lutetiensis	2,005,648	Contig	GCF_014847295.1
Streptococcus lutetiensis	1,941,208	Scaffold	GCF_015670875.1
Streptococcus lutetiensis	1,971,124	Chromosome	GCF_016403165.1
Streptococcus lutetiensis	1,793,521	Complete	GCF_016725165.1
Streptococcus lutetiensis	1,880,658	Contig	GCF_018365495.1

Streptococcus lutetiensis	1,847,904	Contig	GCF_018379155.1
Streptococcus lutetiensis	1,892,899	Contig	GCF_018499085.1
Streptococcus lutetiensis	1,858,518	Scaffold	GCF_018499095.1
Streptococcus lutetiensis	1,884,990	Scaffold	GCF_018499145.1
Streptococcus lutetiensis	1,823,989	Contig	GCF_018499195.1
Streptococcus lutetiensis	1,768,029	Contig	GCF_018499235.1
Streptococcus lutetiensis	2,092,202	Scaffold	GCF_018499245.1
Streptococcus lutetiensis	1,831,521	Contig	GCF_018499285.1
Streptococcus lutetiensis	1,824,049	Contig	GCF_018499295.1
Streptococcus lutetiensis	1,809,342	Contig	GCF_018499325.1
Streptococcus lutetiensis	1,822,427	Contig	GCF_018499345.1
Streptococcus lutetiensis	1,843,148	Contig	GCF_018499365.1
Streptococcus lutetiensis	1,909,663	Contig	GCF_018499425.1
Streptococcus lutetiensis	1,900,102	Contig	GCF_018499485.1
Streptococcus lutetiensis	1,850,252	Contig	GCF_018499535.1
Streptococcus lutetiensis	1,864,887	Contig	GCF_018499565.1
Streptococcus lutetiensis	1,793,719	Contig	GCF_018499605.1
Streptococcus lutetiensis	1,842,783	Contig	GCF_018499615.1
Streptococcus lutetiensis	1,791,563	Contig	GCF_018499625.1
Streptococcus lutetiensis	1,823,680	Contig	GCF_018499675.1
Streptococcus lutetiensis	1,900,273	Contig	GCF_018499705.1
Streptococcus lutetiensis	1,957,884	Scaffold	GCF_018499715.1
Streptococcus lutetiensis	1,835,103	Contig	GCF_018499745.1
Streptococcus lutetiensis	1,964,905	Scaffold	GCF_018499755.1
Streptococcus lutetiensis	1,853,606	Contig	GCF_018530985.1
Streptococcus lutetiensis	1,825,671	Contig	GCF_018530995.1
Streptococcus lutetiensis	1,894,183	Contig	GCF_018531035.1
Streptococcus lutetiensis	1,847,329	Contig	GCF_018917365.1
Streptococcus lutetiensis	1,801,956	Contig	GCF_019012505.1
Streptococcus lutetiensis	1,769,561	Contig	GCF_022737755.1
Streptococcus lutetiensis	1,568,145	Contig	GCF_022749235.1
Streptococcus lutetiensis	1,782,787	Contig	GCF_023109735.1
Streptococcus lutetiensis	1,843,934	Contig	GCF_023110015.1
Streptococcus lutetiensis	1,799,065	Scaffold	GCF_026782985.1
Streptococcus lutetiensis	1,827,900	Scaffold	GCF_027670025.1
Streptococcus lutetiensis	1,808,257	Contig	GCF_032481975.1
Streptococcus lutetiensis	1,804,985	Contig	GCF_032482915.1
Streptococcus lutetiensis	1,697,986	Contig	GCF_032567455.1
Streptococcus lutetiensis	1,883,376	Contig	GCF_032571215.1
Streptococcus lutetiensis	1,780,685	Contig	GCF_032571965.1
Streptococcus lutetiensis	1,950,307	Contig	GCF_032582965.1
Streptococcus lutetiensis	1,869,615	Contig	GCF_036302885.1
Streptococcus lutetiensis	1,966,581	Complete	GCF_900475065.1
Streptococcus lutetiensis	1,793,521	Complete	GCF_900475675.1
Streptococcus lutetiensis	1,852,036	Complete	GCF_900635935.1
Streptococcus lutetiensis	1,807,172	Complete	GCF_901543605.1
Streptococcus lutetiensis	1,849,037	Contig	GCF_901543635.1
Streptococcus lutetiensis	1,924,589	Contig	GCF_902167605.1
Streptococcus lutetiensis	1,948,071	Scaffold	GCF_902363115.1

Streptococcus lutetiensis	1,845,251	Contig	GCF_905205905.1
Streptococcus lutetiensis	1,849,765	Contig	GCF_905236995.1
Streptococcus lutetiensis	1,854,024	Contig	GCF_905237015.1
Streptococcus lutetiensis	1,854,488	Contig	GCF_905237035.1
Streptococcus lutetiensis	1,902,765	Contig	GCF_905237045.1
Streptococcus lutetiensis	1,812,536	Contig	GCF_937921055.1
Streptococcus lutetiensis	1,686,264	Contig	GCF_958415445.1
Streptococcus lutetiensis	1,759,286	Contig	GCF_958436575.1
Streptococcus lutetiensis	1,911,602	Contig	GCF_958448945.1
Streptococcus lutetiensis	1,719,716	Contig	GCF_959024205.1
Streptococcus lutetiensis	1,777,615	Contig	GCF_959026975.1
Streptococcus lutetiensis	1,599,128	Contig	GCF_959606415.1
Streptococcus lutetiensis	1,885,574	Contig	GCF_963558765.1
